# Cytogenetic analysis of five Ctenidae species (Araneae): detection of heterochromatin and 18S rDNA sites

**DOI:** 10.3897/CompCytogen.v11i4.10620

**Published:** 2017-09-14

**Authors:** Matheus Pires Rincão, João Lucas Chavari, Antonio Domingos Brescovit, Ana Lúcia Dias

**Affiliations:** 1 Laboratory of Animal Cytogenetics; Department of General Biology, CCB, Universidade Estadual de Londrina. Rodovia Celso Garcia Cid, PR 445, km 380, Londrina-Brasil; 2 Special Laboratory of Biological Collections, Instituto Butantan, São Paulo, Brazil

**Keywords:** C-banding, FISH, fluorochrome, meiosis, spider cytogenetics, sex chromosomes

## Abstract

The present study aimed to cytogenetically analyse five Ctenidae species *Ctenus ornatus* (Keyserling, 1877), *Ctenus medius* (Keyserling, 1891), *Phoneutria nigriventer* (Keyserling, 1891), *Viracucha andicola* (Simon, 1906), and *Enoploctenus cyclothorax* (Philip Bertkau, 1880), from Brazil. All species presented a 2n♂ = 28 except for *V. andicola*, which showed 2n♂ = 29. Analysis of segregation and behavior of sex chromosomes during male meiosis showed a sex chromosome system of the type X_1_X_2_0 in species with 28 chromosomes and X_1_X_2_X_3_0 in *V. andicola*. C banding stained with fluorochromes CMA_3_ and DAPI revealed two distributions patterns of GC-rich heterochromatin: (i) in terminal regions of most chromosomes, as presented in *C. medius*, *P. nigriventer*, *E. cyclothorax* and *V. andicola* and (ii) in interstitial regions of most chromosomes, in addition to terminal regions, as observed for *C. ornatus*. The population of Ubatuba (São Paulo State) of this same species displayed an additional accumulation of GC-rich heterochromatin in one bivalent. Fluorescent in situ hybridization revealed that this bivalent corresponded to the NOR-bearing chromosome pair. All analyzed species have one bivalent with 18S rDNA site, except *P. nigriventer*, which has three bivalents with 18S rDNA site. Karyotypes of two species, *C. medius* and *E. cyclothorax*, are described for the first time. The latter species is the first karyotyped representative of the subfamily Acantheinae. Finally, 18S rDNA probe is used for the first time in Ctenidae at the present study.

## Introduction

Ctenidae is a family of Araneae distributed throughout the tropical region of the planet (World Spider Catalog 2017). This family includes wandering and nocturnal spiders, with some species of medical interest, such as those of the genus *Phoneutria* Perty, 1833 (Ministério da Saúde 2017). Ctenidae is divided into five subfamilies, namely Acanthocteninae, Viridasiinae, Cteninae, Calocteninae, and Acantheinae (Silva-Dávila 2003; Polotow and Brescovit 2014). Although ctenids are of great ecological and medical importance, studies on their cytogenetics are scarce (Table 1) and cytogenetic data for the last two subfamilies are not available to date.

**Table 1.** Cytogenetic data of Ctenidae species, updated from Araujo et al. (2014), including the data of present study. NOR = nucleolus organizer region.

Species	Karyotype (♂)	NORs		Reference
		Silver Nitrate	detection of 18S rDNA	
**Acantheinae**				
*Enoploctenus cyclothorax* (Bertkau, 1880)	28, X_1_X_2_0		2	Present study
**Acanthocteninae**				
*Nothroctenus* sp.	29, X_1_X_2_X_3_0			Araujo et al. 2014
*Viracucha andicola* (Simon, 1906)	29, X_1_X_2_X_3_0	4		Araujo et al. 2014
			2	Present study
**Cteninae**				
*Anahita fauna* Karsch, 1879	29, X_1_X_2_X_3_0			Chen, 1999
*Ctenus indicus* (Gravely, 1931)	28, X_1_X_2_0	4		Kumar et al. 2016
*Ctenus medius* Keyserling, 1891	28, X_1_X_2_0		2	Present study
*Ctenus ornatus* (Keyserling, 1877)	28, X_1_X_2_0	2		Araujo et al. 2014
*Ctenus* sp.	28, X_1_X_2_0			Araujo et al. 2014
*Parabatina brevipes* (Keyserling, 1891)	28, X_1_X_2_0			Araujo et al. 2014
*Phoneutria nigriventer* (Keyserling, 1891)	28, X_1_X_2_0	2		Araujo et al. 2014
			6	Present study
**Viridasiinae**				
*Asthenoctenus borelli* Simon, 1897	22, X_1_X_2_0			Araujo et al. 2014

Three karyotypes have been observed in the family: (i) 2n♂ = 22 (20 + X_1_X_2_0); (ii) 2n♂ = 28 (26 + X_1_X_2_0); and (iii) 2n♂ = 29 (26 + X_1_X_2_X_3_0) (Table 1). The sex chromosome systems (SCS) in spiders are considered highly diverse by many authors (Král et al. 2006; 2011; Araujo et al. 2012) ranging from simple systems, such as XY or X0, to multiple SCS as X_n_Y_n_ or X_n_0 (Araujo et al. 2017). Based on findings in a specimen of *Ctenus ornatus* (Keyserling, 1877) Araujo et al. (2014) suggested that the X_1_X_2_X_3_0 system in Ctenidae, might have arisen from a supernumerary chromosome and, according to literature evidence, this system arose repeatedly in the evolutionary history of Entelegynae and its conversion into the X_1_X_2_0 system and vice-versa is a recurring event. Bole-Gowda (1952) also suggested the involvement of a supernumerary element in the origin of the X_3_ chromosome in Sparassidae species. Other hypotheses on the conversion of a X_1_X_2_0 into a X_1_X_2_X_3_0 were also proposed by some authors (Pätau 1948; Postiglioni and Brum-Zorrilla 1981; Parida and Sharma 1986). The conversion of a X_1_X_2_X_3_0 into a X_1_X_2_0 was firstly proposed in the spider genus *Malthonica* Simon, 1898 (Agelenidae) by Král (2007), suggesting that tandem fusions occurred in this process.

Chromosome banding techniques, as identification of nucleolus organizer regions (NORs) using silver nitrate impregnation, have been performed in Ctenidae. Araujo et al. (2014) found a single terminal NOR on one autosomal pair in *C. ornatus* and *Phoneutria nigriventer* (Keyserling, 1891), and on two pairs in *Viracucha andicola* (Simon, 1906). Kumar et al. (2016) also detected NORs on two autosomal pairs in *Ctenus indicus* (Gravely, 1931). However, molecular cytogenetic studies are scarce in spiders. There have been only five studies about distribution of some sequences using fluorescence *in situ* hybridization (FISH): location of 18S rDNA sites in *Wadicosa fidelis* (O. Pickard-Cambridge, 1872) (Lycosidae) (Forman et al. 2013) and *Brachypelma albopilosum* Valerio, 1980 (Theraphosidae) (Král et al. 2013); 5S rDNA sites in *Oxyopes sertatus* L. Koch, 1878 (Oxyopidae) (Suzuki and Kubota 2011); mapping of silk genes in *Latrodectus hesperus* Chamberlin & Ivie, 1935 and *Latrodectus geometricus* C. L. Koch, 1841 (Theridiidae) (Zhao et al. 2010); and ocurrence of telomeric repeats in *Brachypelma albopilosa* Valerio, 1980 (Vítková et al. 2005).

Considering the great importance of ctenids and the scarcity of cytogenetic studies in the group, our study analyzed the mitotic and meiotic chromosomes of five species of this family. To understand better the karyotype structure in this group of spiders, we evaluated the behavior of sex chromosomes, heterochromatin composition/distribution pattern, and the location of 18S rDNA sites.

## Material and methods

### Specimen deposition

Adults and juveniles of five ctenid species from different collection sites in Brazil were analyzed, as listed in Table 2. Specimens were deposited in the arachnological collection of the Laboratório Especial de Coleções Biológicas at Instituto Butantan (IBSP, curator A. D. Brescovit), São Paulo/SP (São Paulo state), Brazil.

**Table 2.** List of collected species, with the number of the individuals, collection sites, and voucher numbers. PR = Paraná State. SP = São Paulo State.

**Species**	**Individuals (♂)**	**Collection Site**	**Voucher Number**
*Ctenus medius*	5	Londrina (23°19'37.5"S, 51°12'13.4"W), PR	166439, 167462, 167463, 167466, 167490
*Ctenus ornatus*	11	Londrina (23°19'37.5"S, 51°12'13.4"W), PR	166426–166430, 166440–166442, 166449, 166458–166459
	9	Céu Azul (25°09'15.8"S, 53°50'42.1"W), PR	166399–166401, 167467–167470, 167476–167477
	2	Foz do Iguaçu (25°37'41.2"S 54°27'47.2"W), PR	166416, 167465
	4	Ubatuba (23°24'14.3"S 45°03'54.0"W), SP	166453-166454, 167402, 167406
*Enoploctenus cyclothorax*	3	Céu Azul (25°09'15.8"S, 53°50'42.1"W), PR	166397, 166398, 166407
*Phoneutria nigriventer*	5	Londrina (23°19'37.5"S, 51°12'13.4"W), PR	166441, 167407, 167489, 167494, 167495
	1	Céu Azul (25°09'15.8"S, 53°50'42.1"W), PR	166412
	1	Foz do Iguaçu (25°37'41.2"S 54°27'47.2"W), PR	167405
*Viracucha andicola*	6	Londrina (23°19'37.5"S, 51°12'13.4"W), PR	166434, 166445, 166447, 167398–167400
	2	Céu Azul (25°09'15.8"S, 53°50'42.1"W), PR	166411, 166413

### Chromosome preparations and banding

Chromosomal preparations were obtained according to Araujo et al. (2008), with some modifications as follows. After the fixation, testes were dissociated in a drop of 60% acetic acid on the surface of a microscope slide and covered with a coverslip, pressed and immersed in liquid nitrogen to allow the removal of the coverslip. The diploid number was determined by counting 30 meiotic and mitotic cells. The morphology of chromosomes was classified according to Levan et al. (1964), using the MicroMeasure version 3.3 software (Reeves and Tear 2000). To determine the heterochromatin location and its composition, the slides were submitted to C-banding following Sumner (1972) and subsequently stained with base-specific fluorochromes, chromomycin A_3_ (CMA_3_) and 4’, 6-diamidino2-phenilindole (DAPI), according to the procedure described by Schweizer (1980).

### 18S rDNA probe generation

Genomic DNA of *C. ornatus* was extracted using a standard phenol/chloroform procedure (Sambrook and Russell 2006). A polymerase chain reaction (PCR) was performed with the primers of 18S rDNA, forward: CGAGCGCTTTTATTAGACCA and reverse: GGTTCACCTACGGAAACCTT, as described by Forman et al. (2013). Another pair of primers was designed in the Primer3Plus software (Untergasser et al. 2007) to allow the complete amplification of the 18S rDNA fragment, forward: TCTGTCTCGTGCGGCTAAAC and reverse: GATCCATTGGAGGGCAAGTC. The PCR reaction contained diluted genomic DNA, *Taq* buffer, 0.8 mM dNTP mix, 4 mM MgCl_2_, 5 pmol of each primer, and 2.5 U of *Taq* polymerase (Invitrogen) for a reaction of 25 µl. The amplification was performed with an initial denaturation of 2 min at 94 °C, followed by 40 cycles of 1 min at 94 °C, 1 min at 60 °C, and 5 min at 72 °C until completion. The 18S rDNA was purified by agarose gel using the Pure Link-Quick Gel Extraction Kit (Invitrogen). The DNA fragment generated by the pair of primers described by Forman et al. (2013) was cloned using the kit pGEM-T Easy Vector System (Promega) in a suitable strain of *Escherichia coli* (TOP 10) and the insert was sequenced by the ABI-Prism 3500 Genetic Analyzer (Applied Biosystems).

The sequence was analyzed using the free software BioEdit, version 7.2.5 (Hall 2013). The rDNA sequence of 1280 pb, obtained from *C. ornatus*, was submitted to BLASTN (Altschul et al., 1990) in the National Center for Biotechnology Information (NCBI) database, through web site (http://www.ncbi.nlm.nih.gov/blast), to verify the homology with sequences of 18S rDNA from spiders and demonstrated 99% of homology with *Phoneutria fera* Perty, 1833 (accession KY016373.1) in the GenBank. The sequence was deposited on NCBI, accession KT698160.1.

### Fluorescence *in situ* hybridization

The 18S rDNA sites were identified using the FISH technique according to Pinkel et al. (1986) and Gouveia et al. (2013), with the following modifications. After dehydration, the slides were treated with formamide 15%/SSC for 10 min and subsequently in pepsin (0.005 mg/mL) for 20 min. Probes were labeled with the Dig-Nick Translation kit (Invitrogen) and detected by the monoclonal anti-digoxigenin antibody conjugated to rhodamine (Roche Applied Science, Indianapolis, IN). Preparations were counter-stained with DAPI. In the Ubatuba *C. ornatus* population, the slides were stained after a FISH procedure with CMA_3_ and DAPI to visualize the association between 18S rDNA sites and GC-rich blocks. Finally, the slides were analyzed in an epifluorescence microscope (Leica DM 2000), equipped with a digital camera Moticam Pro 282B. The images were captured using the Motic Images Advanced software, version 3.2.

## Results

**Figure 1.**
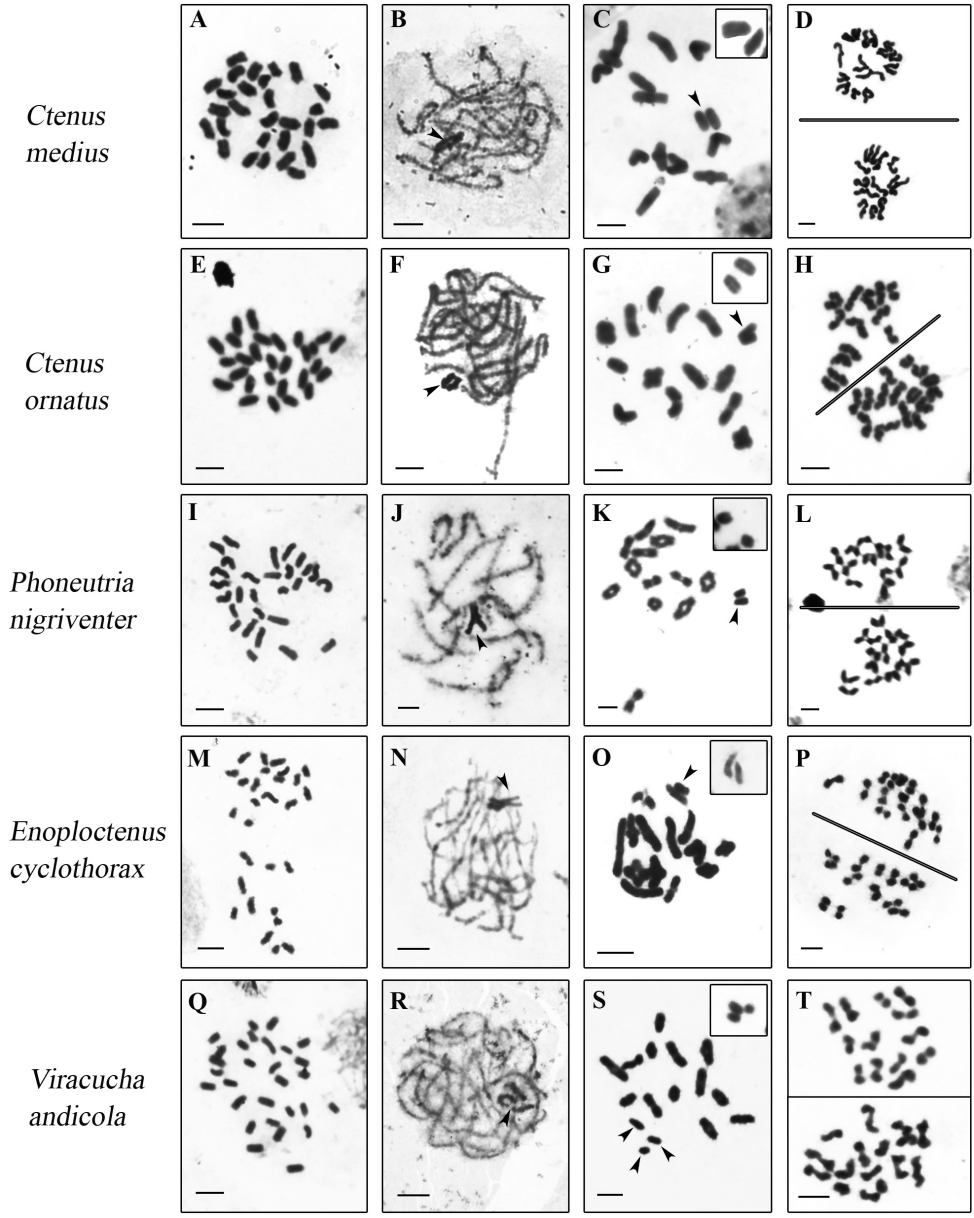
Male mitotic and meiotic cells of Ctenidae species stained with Giemsa. Boxes – X chromosomes without association (**C, G, K, O**), and with association (**S**). *C. medius* (**A–D**), *C. ornatus* (**E–H**), *P. nigriventer* (**I–L**), *E. cyclothorax* (**M–P**), *V. andicola* (**Q–T**). The arrowheads show sex chromosomes. Mitotic metaphases with 2n = 28 (**A, E, I, M**) and 2n =29 (**Q**). Pachytene cells **(B, F, J, N, R**) with positively heteropycnotic sex chromosomes. Diakinesis cells (**C, G, K, O, S**), note parallel association of two X chromosomes (**C**, **G**, **K**, **O**) or three X chromosomes without association (**S**). Metaphase II cells with n = 13 and n = 13 + X_1_X_2_ **(D, H, L, P)** and n = 13 and n = 13 + X_1_X_2_X_3_ **(T)**. Bar = 10 µm.

*Ctenus ornatus*, *Ctenus medius* Keyserling, 1891, *Phoneutria nigriventer*, and *Enoploctenus cyclothorax* (Bertkau, 1880) exhibited 2n♂ = 28, as observed in mitotic metaphases (Fig. 1A, E, I, M), whereas *Viracucha andicola* presented 2n♂ = 29 (Fig. 1Q). All chromosomes were identified in metaphases II as acrocentric (Fig. 1D, H, L), except for *E. cyclothorax* and *V. andicola*, in which it was difficult to determine accurately the morphology of all chromosomes (Fig. 1P, T).

At male diakinesis 13 bivalents in all species were found and two univalent X in parallel association in the species with 28 chromosomes (Fig. 1C, G, K, O) and three univalent X in the species with 29 chromosomes (Fig. 1S). Three sex chromosomes in *V. andicola* showed parallel association (Fig. 1S-box). In some plates at pachytene and diplotene X are not associated in species with the two X chromosomes (Fig. 1C, G, K, O-boxes). Species with 2n♂ = 28 showed metaphases II with 13 and 15 chromosomes (Fig. 1D, H, L, P), and species with 2n♂ = 29 showed cells with 13 and 16 chromosomes (Fig. 1T), that confirm sex chromosome systems of the types X_1_X_2_0 and X_1_X_2_X_3_0, respectively. In species with 28 chromosomes, two positively heteropycnotic bodies were observed in pachytene stage (Fig. 1B, F, J, N) and *V. andicola* exhibited three positive heteropycnotic bodies (Fig. 1R), identified as the sex chromosomes.

**Figure 2.**
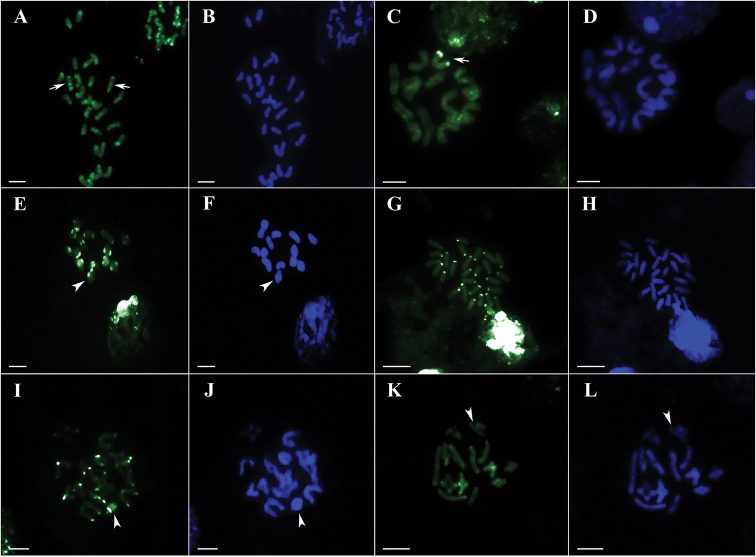
Ctenidae male mitotic and meiotic cells, C-banding and staining with base-specific fluorochromes CMA_3_ (**A, C, E, G, I, K**) and DAPI (**B, D, F, H, J, L**). Arrowhead - X chromosomes. **A, B** mitotic metaphase of *Ctenus ornatus*, 28 chromosomes, arrow – interstitial CMA_3_^+^ region **C, D** diakinesis of *C. ornatus*, Ubatuba population, arrow – bivalent with large CMA_3_^+^ block **E, F** diakinesis of *C. medius* **G, H** mitotic metaphase of *Phoneutria nigriventer*, 2n=28 **I, J** diakinesis of *Viracucha andicola* **K, L** diakinesis of *Enoploctenus cyclothorax*. Bar = 10 µm.

*Ctenus ornatus* presented interstitial and terminal CMA_3_^+^ bands (Fig. 2A). Nevertheless, the population of Ubatuba (São Paulo state) presented an additional large terminal CMA_3_^+^ block in a bivalent (Fig. 2C). In *C. medius* (Fig. 2E), *P. nigriventer* (Fig. 2G), *V. andicola* (Fig. 2I), and *E. cyclothorax* (Fig. 2K), all populations showed only CMA_3_^+^ terminal blocks. Karyotypes contained no DAPI^+^ blocks (Fig. 2B, D, F, H, J, L).

**Figure 3.**
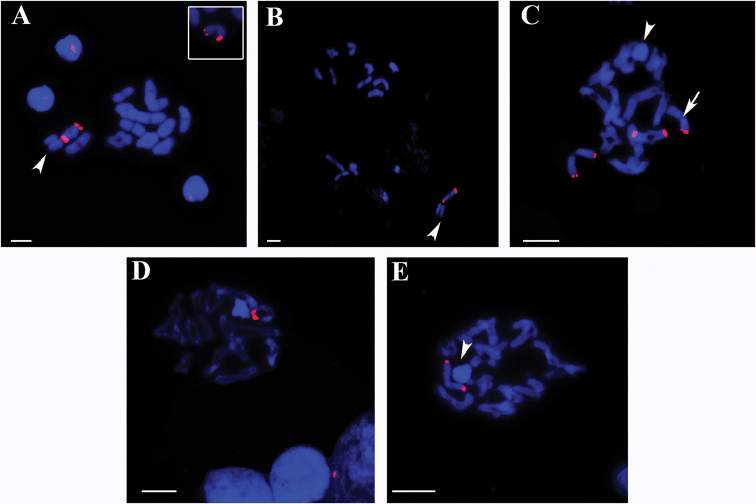
Ctenidae male meiotic cells, FISH with rDNA 18S probe. Arrowhead - sex chromosomes. **A** diakinesis of *Ctenus ornatus*: in the box the bivalent with size heteromorphism of 18S rDNA sites **B** diakinesis of *Ctenus medius* **C** diakinesis of *Phoneutria nigriventer*: arrow - bivalent with 18S rDNA sites in only one of the chromosomes **D** diplotene of *Viracucha andicola* **E** diplotene of *Enoploctenus cyclothorax*. Bar = 10 µm.

The FISH revealed one bivalent with 18S rDNA site in *C. ornatus* (Fig. 3A), *C. medius* (Fig. 3B), *V. andicola* (Fig. 3D), and *E. cyclothorax* (Fig. 3E). *C. ornatus* presented size polymorphism of the 18S rDNA site (Fig. 3A-box). *P. nigriventer* showed three bivalents exhibiting 18S rDNA site; however, one of these bivalents presented site only in one chromosome (Fig. 3C).

**Figure 4.**
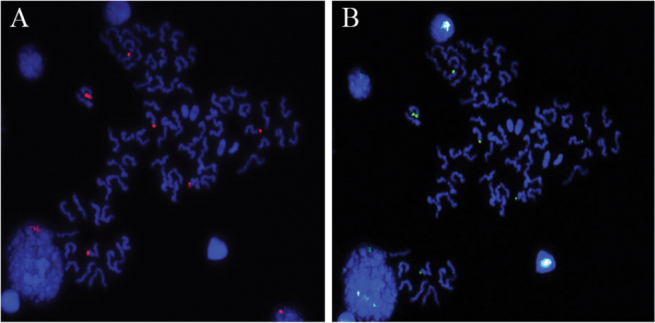
Chromosomes of *Ctenus ornatus*, Ubatuba/São Paulo state. **A** Metaphase II, FISH with rDNA 18S probe **B** sequential staining with DAPI/CMA_3_ in the same metaphase II, showing association between sites of GC-rich heterochromatin and rDNA 18S regions. Note the presence of more than one metaphase II. Bar = 10 µm.

Metaphase II of *C. ornatus* from the Ubatuba population submitted to FISH and subsequently to CMA3/DAPI also revealed that CMA^+^ sites with higher accumulation of GC-rich heterochromatin are co-localized to the sites carrying 18S rDNA (Fig. 4).

## Discussion

The conventional analysis showed diploid number, chromosomal morphology, sex chromosome system and meiotic behavior of five Ctenidae species. The present study presents the first data for Acantheinae, increasing to four the number of ctenid subfamilies with cytogenetic data (Table 1), and the first cytogenetic study in *C. medius* and *E. cyclothorax*. In Ctenidae, the diploid number variation occurs basically due to the differences in SCS: species with 2n♂ = 28 exhibit a SCS of the type X_1_X_2_0, whereas species with 2n♂ = 29 have the type X_1_X_2_X_3_0. Only *A. borellii* (Viridasiinae) presents 2n♂ = 22, with SCS of the type X_1_X_2_0 (Chen 1999, Araujo et al. 2014, Kumar et al. 2016).

The parallel association between sex chromosomes during male meiosis is a common pattern observed in Entelegynae (Král et al. 2011; Araujo et al. 2012), and also found in Ctenidae (Chen 1999; Araujo et al. 2014; Kumar et al. 2016). Forman et al. (2013) observed absence of sex chromosome pairing in some plates of *Wadicosa fidelis*. They proposed that it might be due to chromosome preparation. A similar situation may have occurred in species analyzed in this study.

We observed two distinct distribution patterns of the GC-rich heterochromatin: (i) bands distributed in terminal regions of most chromosomes, as presented in *C. medius*, *P. nigriventer*, *E. cyclothorax* and *V. andicola*; and (ii) bands present in interstitial regions of most chromosomes, in addition to the terminal regions, as observed for *C. ornatus*. The first pattern could arise by dispersion of heterochromatin due to contact of chromosomes during their polarization of Rabl in mitosis or during bouquet orientation at the early prophase I as described by Schweizer and Loidl (1987). The second pattern could arise by occurrence of chromosomal rearrangements (Schweizer and Loidl 1987) or by spreading of the heterochromatin by transposable elements, as proposed for grasshopper (Rocha et al. 2015). Furthermore, despite the few species studied, GC-rich blocks seem to be common in entelegyne spiders (Araujo et al. 2005; Ramalho et al. 2008, Chemisquy et al. 2008). They were also found in Ctenidae species in the present study. The heterochromatin distribution also allowed to distinguish *C. ornatus* from Ubatuba population of other *C. ornatus* populations here analyzed.

The present study revealed a massive accumulation of GC-rich heterochromatin associated with 18S rDNA site in *C. ornatus* from Ubatuba. Association of GC-rich heterochromatin with NORs is common in many animal groups, for example in fishes (Ferro et al. 2001) and amphibians (Schmid 1980). In spiders, this association has been reported in *Nephilingys cruentata* (Araneidae) (Araujo et al. 2005).

Another characteristic observed in *C. ornatus* was the size heteromorphism of 18S rDNA sites. This can be explained by unequal crossing, which causes a greater accumulation of rDNA cistrons in one of the homologous chromosomes, as described by Ferro et al. (2001) and Teribele et al. (2008) in fish species. A similar situation may have occurred in *P. nigriventer*, very small 18S rDNA sites could exhibit low fluorescence, making detection difficult.

In Ctenidae, NOR in one bivalent seems to be the most commonly observed pattern. Only *P. nigriventer* presented more rDNA sites. This finding differs from Araujo et al. (2014), who observed only one chromosome pair carrying NOR in the same species using the silver nitrate impregnation that identifies only transcriptionally active sites. Specimens of *V. andicola* showed a single NOR as revealed by the FISH analysis. By contrast, the data exhibited by Araujo et al. (2014) showed NORs in two chromosome pairs, which could indicate an interpopulation variation, however the authors analyzed only one specimen, which hinders a more accurate study.

The present study brings new cytogenetic information and first FISH data for Ctenidae providing valuable contribution to the knowledge on karyotypes in this family.
